# 

**Published:** 1886-01

**Authors:** 


### List of Original Contributors to Vol. VII.

| | |
|---|---|
| ANDERSON, C. L | D. D. S. |
| BARRETT, W. C | M. D., D. D. S., M. D. S. |
| BIRDSALL, HERBERT A | D. D. S. |
| BLACK, G. V | M. D., D. D. S. |
| BODECKER, C. F. W | D. D. S., M. D. S. |
| BRISTOL, L. W | DR. |
| CHANCE, GEO. H | D. D. S. |
| CHITTENDEN, C. S | D. D. S., L. D. S. |
| CLOWES, J. W | D. D. S. |
| CURTIS, G. L | D. D. S. |
| EAMES, W. H | D. D. S. |
| FARRAR, J. N | M. D., D. D. S. |
| FRANCIS, C. E | D. D. S., M. D. S. |
| FRIEDRICHS, GEO. J | M. D., D. D. S. |
| HARLAN, A. W | M. D., D. D. S. |
| HEITZMANN, CARL | M. D. |
| HOPKINSON, B. MERRILL | D. D. S., M. D. |
| HOWARD, F. E | M. D. S. |
| HOWLAND, SAMUEL F | D. D. S. |
| INGERSOLL, L. C | A. B., D. D. S. |
| JENKINS, CHAS | D. D. S. |
| LAND, C. H | DR. |
| MILLER, W. D | A. B., PH.D., D. D. S. |
| MOODY, J. D. | D. D. S. |
| NEWKIRK, GARRETT | M. D. |
| NILES, EDW. S | D. M. D. |
| PALMER, S. B | M. D. S. |
| PARMELE, GEO. L | M. D., D. M. D. |
| SOUTHWORTH, S. S | DR. |
| SPALDING, J. H | D. D. S. |
| STARR, ALFRED R | M. D., D. D. S. |
| SWAIN, EDGAR D | D. D. S. |
| SWASEY, JAMES A | D. D. S. |
| TALBOT, E. S | M. D., D. D. S. |
| TRUEMAN, W. H | D. D. S. |
| VANDERPANT, LAWRENCE | L. D. S. |
| VAN MARTER, J. G | A. M., D. D. S. |
| WITZEL A | DR. |

### INDEX

TO THE

### INDEPENDENT PRACTITIONER.

### VOLUME VII.

ORIGINAL COMMUNICATIONS.

**A**pplication of and Elements of Success in Crown Work. C. L. Anderson. 465
Aqua Calcis. George J. Friedrichs.................................. 235

**C**alcific Deposits in Tooth Pulps. Edgar D. Swain....................... 281
Caries and Necrosis of the Jaws. J. H. Martindale....................... 674
Chicago College of Dental Surgery. James A. Swasey...................... 245
Cocoaine, About. C. E. Francis...... .................................. 356
Crown Work and English Tube Teeth. LawrenceVanderpant ........... 20

**D**. D. S. or M. D.; Which? Geo. H. Chance................................ 343
Density of Teeth as Influenced by the Food and by the Administration of Lime Salts. W. D. Miller........................................... 597
Dental Chemistry. S. B. Palmer........................................ 241
Dental Phosphorénèses, or Caries. Edward S. Niles.................... 1
Duties of a Dentist to Himself and Others, The. G. L. Curtis............ 130

**E**ditors and Editing. Garrett Newkirk................................ 81
Exostosis, or Dental Osteoma. W. C. Barrett.............................. 409
Extraction of Teeth for Parturient Women, The. Garrett Newkirk...... 18

**F**urther Evidence of Prehistoric Dentistry. J. G. Van Marter........... 57

**G**elatine-forming Micro-organisms. G. V. Black.......................... 546
Golden. J. W. Clowes..... ........... ........................ 612

**H**istorical Reminiscences. C. S. Chittenden............................. 65
Hydro-Carbon Furnaces for Dental Operations. C. H. Land............. 14

**I**nflammation of Dentine (Eburnitis). Carl Heitzmann and C. W. F. Bodecker 120
Irregularities of the Teeth and their Treatment. F. E. Howard.......... 359

**L**oculosis Alveolaris. J. N. Farrar..................................... 457

**N**ew System of Restoring Badly Decayed Teeth by means of an Enameled Metallic Coating, A. C. H. Land..................................... 407
Notes on the Decay of Human Teeth. W. D. Miller.................... 533

On Certain Fermentative Processes in the Alimentary Canal, and the Micro-organisms by which they are Produced. W. D. Miller............61, 113
On Certain Gas-forming Bacteria of the Alimentary Canal, their Fate in the Stomach, and their Reaction on Different Foods. W. D. Miller....... 169

Pain Obtunders. A. W. Harlan.................................... 475
Perils of Practice, The. Geo. L. Parmele......................... 73
Personal Recollections of a Dentist of the Early Days. L. W. Bristol....9, 683
Pocket Disease of the Alveolus. J. N. Farrar.......................... 172
Popular Appreciation of Dentistry. L. C. Ingersoll...................... 542
Professional Courtesies in Connection with what are Commonly Considered Failures in Dental Practice. C. E. Francis.............................. 538
Pyorrhœa Alveolaris. Alfred R. Starr.............................397, 661

Retrospective Glance, A. Herbert A. Birdsall........................... 292

Sanitas. E. S. Talbot.................................................. 19
Sanitas Oil. A. W. Harlan.............................................. 184
Sanitas Oil. E. S. Talbot.............................................. 296
Scythian Dentistry. W. H. Eames....................................... 290
Setting Teeth in Artificial Sockets. S. S. Southworth.................. 478
Some Thoughts and Experiments upon Erosion. Edgar D. Swain.....178, 225
Systematic Study. J. D. Moody.......................................... 480

Teeth with Exposed Pulps. Devitalization and Subsequent Treatment. B. Merrill Hopkinson............................................... 380
Teeth with Exposed Pupls. Garrett Newkirk............................. 548
Testing the Power of Antiseptics W. D. Miller.......................... 288
Tin and Gold Combined J. H. Spaulding.................................. 77
Tooth Crowns. Samuel F. Howland........................................ 137

Uses of Ferrules in Regulating Teeth, The. J. N. Farrar................ 337

Verrier's and Schiltsky's Continuous Gum Methods. A. Witzel........... 79
Visit to Foreign Dental Schools, and other Observations. A. W. Harlan ..............................................229, 393, 605

Wail of the Second Bicuspid. Chas. Jenkins............................. 133

### REPORTS OF SOCIETIES.

American Academy of Dental Science .................................. 98
American Dental Association.....................22, 83, 482, 550, 616, 689
American Dental Society of Europe...................................... 31

Central Dental Association of Northern New Jersey.................253, 299

Dr. Wilhelm Herbst's Visit to America.................................. 433

First District Dental Society of New York.........38, 139, 190, 263, 627, 693

Herbst Clinics in America, The.........................505, 574, 637, 701

Illinois State Dental Society..........305, 362, 445

Louisiana State Dental Society..........198, 247

Meeting of the German Naturalists and Physicians at Berlin.......... 696

New Orleans Odontological Society.......... 260
New York Odontological Society..........94, 148, 194, 303, 372

Pennsylvania State Dental Society..........489, 561, 630

Southern Dental Association..........496, 567

### EDITORIALS.

A. D. A Meeting, The.......... 449
Abbot, Dr. F. P.......... 651
American Dental Association, The.......... 323
And yet Another Journal.......... 710
Another Dental Journal.......... 652
Another Specific.......... 105
Apologetic.......... 45
Another Apology.......... 524
Apologizing Again.......... 653
Are Micro-organisms Scavengers or the Producers of Pathological Conditions? 449
Attention.......... 524

Cocaine.......... 270
Cogswell's Disk Carrier.......... 46
Craniotomy.......... 105
Critic, A Muddled.......... 104
Crowded Out.......... 158

"Doctor".......... 650
Donaldson's Pulp Canal Cleansers.......... 159
Dr. Allport's Pyorrhœa Instruments.......... 45
Dr. Miller's Paper on Fermentation.......... 158

End of the Volume.......... 709

Fraudulent Certificates from Germany.......... 652

Greek Terminations.......... 584

Herbst Clinics at Niagara, The.......... 451
Herbst Methods, The.......... 583

Illinois State Dental Society, The.......... 382
Implantations of Teeth.......... 646
International Medical Congress Again.......... 43

Labor Lost.......... 45

Medical Specialties.......... 524

Niagara Meeting, The.................... 379

Our Book Table.................... 711

Place of Meeting, The.................... 206
Post-Graduate Study.................... 325
Prehistoric Art.................... 158
Pulpitis.................... 381

Question of Propriety.................... 156

Scientific Accuracy.................... 268
Scientific Intolerance.................... 523
Scientific Societies.................... 452
Settled at Last.................... 266

"The Dental Profession".................... 269
To Contributors.................... 452
To Junior Dentists, No. 4.................... 99
To Junior Dentists, No 5.................... 319
To Junior Dentists, No 6.................... 374
To Junior Dentists, No. 7.................... 704
To New Subscribers.................... 326
To Our Subscribers.................... 380
To Subscribers.................... 452
Twenty-sixth Annual Meeting of the American Dental Association, The... 519

Underwood Spring Water.................... 159

Visit of Wilhelm Herbst, The.................... 446

Why do Indians Have Such Good Teeth.................... 450

BIBLIOGRAPHICAL.

....................46, 159, 271, 327, 585

CURRENT NEWS AND OPINION.

.....................53, 106, 164, 207, 273, 329, 383, 453, 525, 591, 654, 712

### The Independent Practitioner—Vol. VII.

### PUBLISHED BY DENTISTS FOR DENTISTS.

### Prospectus.

This Journal is published by an association of professional men who have no ulterior objects other than to afford to the profession an independent, outspoken Journal, which shall be the exponent of higher aims and a better practice.

Having no connection with any school, clique, or advertising firm, it will be impartial in its judgment of professional matters, and its appeals for support are directed to those who believe that a liberal profession should have an indeoendent literature.

While not neglecting practical details, it will pay especial attention to those oral diseases that have usually been under the care of the general practitioner, but which more properly belong to the specialty of dentistry.

Its pages will be open to any member of the profession who has anything of interest to communicate, and it invites a free expression of views on all subjects medical or dental.

As each of the gentlemen forming the association is practically a member of the editorial corps, it will have abundant opportunity for collecting all the latest professional news and intelligence.

It will have a body of regular contributors, selected from the very best writers in the profession, and will thus be certain of an abundance of interesting original matter.

It will pay especial attention to reports of the meetings of dental societies, and its aim will be to publish an epitome of all important discussions of professional matters.

It will contain original abstracts and translations of all that is of moment in the foreign journals.

Its profits, if any, will be devoted to its improvement by adding needed illustrations, paying for valuable original communications, and enlarging its size whenever this becomes advisable.

To the accomplishment of the work thus outlined (necessarily relying upon the hearty approval of an appreciative profession), each of the publishers of the Independent Practitioner has pledged his best efforts.

Editorial communications should be addressed to Dr. W. C. Barrett, No. 208 Franklin St., Buffalo, N. Y. Send all business letters to Dr. William Carr, 35 West Forty-Sixth Street, New York.

| | | |
|---|---|---|
| ABBOTT, FRANK....New York. | CARR, WILLIAM..New York. | HILL, O. E..........Brooklyn. |
| BARRETT, W. C.........Buffalo. | DUDLEY, A. M..Salem, Mass. | NORTHROP, A. L.New York. |
| BODECKER, C F. W..New York. | FRANCIS, C. E....New York. | PALMER, S. B......Syracnse. |

